# Supercritical Synthesis of Biodiesel 

**DOI:** 10.3390/molecules17078696

**Published:** 2012-07-23

**Authors:** Juana M. Bernal, Pedro Lozano, Eduardo García-Verdugo, M. Isabel Burguete, Gregorio Sánchez-Gómez, Gregorio López-López, Mathieu Pucheault, Michel Vaultier, Santiago V. Luis

**Affiliations:** 1 Departamento de Bioquímica y Biología Molecular B e Inmunología, Facultad de Química, Regional Campus of International Excellence “Campus Mare Nostrum”, Universidad de Murcia, Campus de Espinardo, E-30100 Murcia, Spain; Email: jmbernalpalazon@um.es; 2 Departamento de Química Inorgánica y Orgánica, Universidad Jaume I, Campus del Riu Sec, E-12071 Castellón, Spain; Email: cepeda@uji.es (E.G.-V.); burguete@uji.es (M.I.B.); luiss@uji.es (S.V.L.); 3 Departamento de Química Inorgánica, Facultad de Química, Regional Campus of International Excellence “Campus Mare Nostrum”, Universidad de Murcia, Campus de Espinardo, E-30100 Murcia, Spain; Email: gsg@um.es (G.S.-G.); gll@um.es (G.L.-L.); 4 Groupe Phoenics, Institut des Sciences Moléculaires, Université Bordeaux 1. CNRS UMR 5255, F33405 Talence cedex, France; Email: m.pucheault@ism.u-bordeaux1.fr (M.P.); m.vaultier@ism.u-bordeaux1.fr (M.V.)

**Keywords:** supercritical fluids, biodiesel, biocatalysis, catalysis, ionic liquids, supported ionic liquids

## Abstract

The synthesis of biodiesel fuel from lipids (vegetable oils and animal fats) has gained in importance as a possible source of renewable non-fossil energy in an attempt to reduce our dependence on petroleum-based fuels. The catalytic processes commonly used for the production of biodiesel fuel present a series of limitations and drawbacks, among them the high energy consumption required for complex purification operations and undesirable side reactions. Supercritical fluid (SCF) technologies offer an interesting alternative to conventional processes for preparing biodiesel. This review highlights the advances, advantages, drawbacks and new tendencies involved in the use of supercritical fluids (SCFs) for biodiesel synthesis.

## 1. Introduction

The depletion of fossil fuels and increasing ecological awareness has led to a search for alternative fuels make from renewable sources such as plant biomass. In 1990 during an exhibition in Paris, a diesel engine was run for the first time for several hours using peanut oil as fuel; however the high viscosity (10–20 times higher than diesel fuel) and low volatility of vegetable oils hindered the generalised use of such fuels in combustion engines. Furthermore, the decomposition of glycerol leads to the formation of the toxic compound acrolein when vegetable oil is combusted in the engine, which further hampers the direct use of vegetable oils as fuels. Replacing the glycerol by short alkyl chain alcohols (e.g., methanol or ethanol) has since provided a low viscous and useful fuel for diesel engine [1].

**Figure 1:**
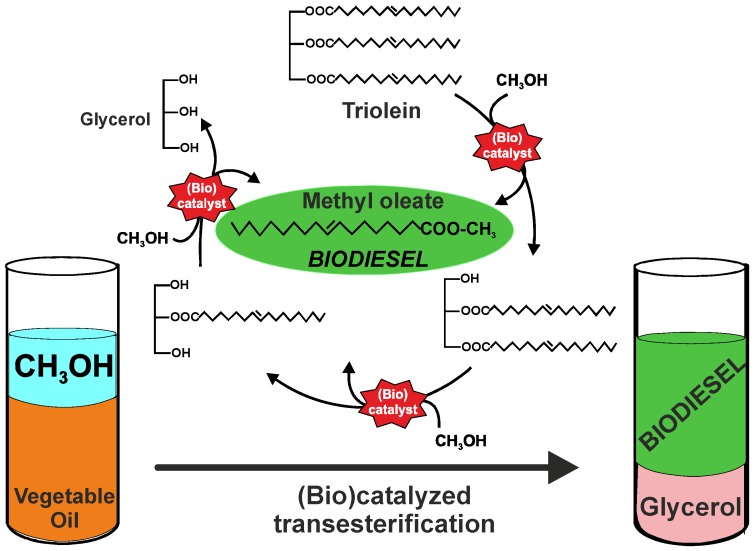
Scheme of the catalyzed transesterification of triglycerides to synthesize biodiesel.

Mixtures of methyl and/or ethyl esters of fatty acids obtained through transesterification of the triacylglycerides contained in plant oils are named biodiesel. This biofuel is so far considered as a good substitute for diesel fuel as it can be used in any compression ignition engine without the need for modifications. Biodiesel is safe, renewable, non-toxic, biodegradable in water, free in sulfur compounds, has a high flash point (>130 °C) and better lubricant properties than diesel. Its use fully complies with the aims and purposes of environmental protection [2]. Biodiesel is usually synthesized through transesterification (also named alcoholysis) of triacylglycerides with methanol, yielding Fatty Acid Methyl Esters (FAMEs) and glycerol as by-product. Because of the reversibility of the reaction, an excess of alcohol is used to shift the equilibrium towards the products side. Three consecutive transesterification reactions are involved in the full conversion of triacylglyceride molecules to biodiesel (see Figure 1).

Catalysis of the transesterification reaction can be roughly divided into two categories, chemical and enzymatic. Chemical catalyzed transesterification reactions can be carried out by either acid or base, in homogeneous or heterogeneous phase, and selection of the conditions depends on the characteristic of the starting triacylglycerides. Basic catalysts are usually preferred to acid catalysts because they provide better reaction rates, lower temperature requirements, and higher conversion efficiencies. NaOH and KOH are classical catalysts, although sodium methoxide derived from the added NaOH is actually the most applied at industrial scale. The protocol involves the dissolution of the catalyst in methanol by vigorous stirring, and mixing the resulting alcohol/catalyst solution with the vegetable oil to give two liquid phases (biodiesel and glycerol) with high yields (>90%) after several hours at 65–90 °C. However, when the free fatty acids (FFAs) content of the triglycerides is higher than 1–2% w/w, basic catalysts can also produce saponification as a side reaction. In such cases, acid catalysts (e.g., H_2_SO_4_) are used, but of large amounts of alcohol (e.g., a molar excess of alcohol over the vegetable oil higher up to 10) and the complete removal of free water are required. Homogeneous catalysts usually give better results than heterogeneous catalysts for biodiesel production. However, many problems are associated with homogeneous catalysis, such as high energy consumption, the formation of unwanted soap by-products because of the presence of FFAs, expensive catalyst separation from the reaction mixture, and specially the generation of large amounts of wastewater during products separation and cleaning. These are clear breakdown points for the sustainability of homogeneous basic catalysis, the usual approach applied at industrial scale for producing biofuel. In contrast, the use of solid acids (e.g., sulfonic acid resins, sulfonic acid-modified silica, *etc.*), or solid bases (e.g., BaO, SrO, MgO, KNO_3_/Al_2_O_3_, *etc.*), as heterogeneous catalysts permits their easy separation from reaction mixture by simple filtration, enabling recycling [3,4].

Biocatalysts represent a completely different approach. As far as all catalyzed reactions are concerned, Nature has always been a source of inspiration for chemists. To transfer the exquisite efficiency shown by enzymes in Nature to chemical processes may well constitute the most powerful toolbox for developing a clean and sustainable chemical industry. Biodiesel synthesis by transesterification and/or esterification using immobilized lipase catalysis is applicable to both refined and raw plant oils, free fatty acids, waste fats from frying, tallow and other waste fats. A small excess of alcohols (e.g., methanol, ethanol or propanol) provides a high biodiesel yields under mild conditions (20–60 °C) [5]. Low water concentrations in the reaction medium can have a positive impact on biodiesel production. In the same way, the conversion of free fatty acids (FFAs) can be much higher than in base catalyzed processes. In this case, both triacylglycerides and FFAs can simultaneously be converted to biodiesel since lipase efficiently catalyzes both transesterification and esterification. Finally, upon completion of the transesterification process, the glycerol (lower phase) is simply separated from the biodiesel (upper phase) and neither product deodorization nor neutralization is required [6].

Probably, the most intriguing feature in the catalytic synthesis of biodiesel is the phase behaviour of the reaction system during the transformation process. The starting materials, triacylglycerides and methanol, are non-miscible, as well as the final products, glycerol and biodiesel (see Figure 1). This phenomenon leads to the low efficiency of immobilized enzymes and their deactivation due to poisoning as results of the adsorption of the continuously formed glycerol by-product onto the support. In this context, both reactor design and reaction medium engineering (e.g., the use of cosolvents, different reaction conditions, *etc.*) are essential approaches to improve the performance of the heterogeneous (bio)catalysts. However, the most important drawback to apply biocatalysts in biodiesel synthetic processes is the high cost of enzymes, making necessary to develop stable and reusable biocatalytic systems for extended operation times [5,6,7].

Several strategies have been developed to overcome these constraints. One example is the sequential addition of methanol in three different doses [8], or its storage by adsorption onto silica gel particles, acting as “microreservoirs” that slowly release the alcohol needed for the transesterification process. In this way, up to 90% biodiesel yield, together with improved catalyst recycling was obtained after 18 h of reaction [9]. Alternative approaches for enzyme immobilization (e.g., encapsulation by sol-gel methods [10], covalent attachment onto magnetic nanoparticles [11], *etc.*) have also been described as improving the biocatalytic efficiency of biodiesel synthesis. Enzyme efficiency (up to 97% yield after 24 h at 50 °C) is also improved by dissolution of the substrates in an organic solvent of medium polarity (e.g., *tert*-butanol), resulting in a one-phase reaction medium and avoiding the direct interaction between the enzyme and pure methanol [12].

Despite being key elements in all chemical processes (e.g., mass-transport, reactions, product separation, *etc.*), volatile organic solvents are also responsible for a not incosiderable part of the environmental impact of the chemical industry. The search for new environmentally benign non-aqueous solvents, or green solvents, which can easily be recovered/recycled, allowing (bio)catalysts to operate efficiently, is a priority for the development of an integral green process of biodiesel synthesis. In this frame, both supercritical fluids (SCFs) and/or ionic liquids, as non-aqueous green solvents, are key targets on the current scientific agenda for biodiesel synthesis. Greenness in chemical processes also concerns the catalysts used, where lipases clearly constitute a powerful green tool for biodiesel synthesis [13]. Consequently, this critical review aims to analyze recent trends and progress in biodiesel synthesis using (bio)catalytic and non-catalytic SCFs technology, with special attention paid to the challenges if a fully clean technology is to be developed for biodiesel production in the near future.

## 2. Supercritical Fluids Technology: Challenges and Limitations

A supercritical fluid (SCF) is a compound, mixture, or element above its critical pressure (*Pc*) and critical temperature (*Tc*), but below the pressure required to condense into a solid [14]. Under such conditions, the densities of both liquid and gas phases become identical, and the distinction between them disappears. The properties of SCFs are frequently described as being intermediate between those of a gas and a liquid. The possibility to manipulate the physical properties of these solvents by simply changing the pressure or temperature is unique to supercritical systems, which show exceptional abilities for extraction, reaction, fractionation and analysis processes. They also determine their environmentally benign character because of the easy way in which they can be fully recovered and reused. However, the key feature of SCFs is their tunability as solvents in response to changes in pressure and temperature, particularly remarkable in the vicinity of the critical point [15]. Table 1 shows the critical parameters of some common fluids used in supercritical conditions. The density of SCFs is highly sensitive to both temperature and pressure; hence, all their density-dependent solvent properties (e.g., dielectric constant, relative permittivity, Hildebrand solubility parameter, *etc.*) may be substantially modified by small changes in pressure or temperature. This provides a potential for controlling (bio)catalyzed reactions by precipitation of a given product, or purification by the selective precipitation of the products.

**Table 1:** Fluids used in near-critical or supercritical conditions for (bio)catalysis and their critical parameters [14].

Fluid	T_c_ (°C)	P_c_ (MPa)	Density (g/L)
Methane (CH_4_)	−82.6	4.60	162
Fluoroform (CHF_3_)	26.2	4.85	516
Carbon dioxide (CO_2_)	31.3	7.29	469
Ethane (C_2_H_6_)	32.3	4.88	203
Sufur hexafluoride (SF_6_)	45.5	3.77	755
Propane (C_3_H_8_)	96.7	4.25	217
Butane (C_4_H_10_)	152.0	3.75	230
Methanol (CH_4_O)	239.6	8.09	272
Ethanol (C_2_H_6_O)	240.9	6.14	276
Acetone (C_3_H_6_O)	235.1	4.70	278
Water (H_2_O)	374.3	22.12	348

Several criteria must be considered before selecting a given SCF as medium for a (bio)catalyzed reaction, including the critical parameters themselves, safety and cost issues. Supercritical carbon dioxide (scCO_2_) is the most popular SCF because of its relatively low critical parameters, low toxicity and non-flammability; furthermore, it is chemically inert under most conditions, has good solvent properties for non-polar solutes, and is considered as a green solvent. Carbon dioxide is clearly a “greenhouse gas”; however, it is massively produced as a by-product at industrial scale (e.g., in ammonia plants), and it is therefore cheap. Furthermore, at atmospheric pressure, CO_2_ is gaseous, which means that simple depressurization is sufficient to separate solutes from scCO_2_, after which it can be pressurized for reuse as a solvent. The solvent power of scCO_2_ can be modified by increasing the bulk density or by adding a modifier (e.g., MeOH, acetone). Co-solvents can therefore be used to increase or reduce polarity, or to enhance the affinity for aromatic substances, although of course, the more co-solvent that is added, the further scCO_2_ moves from being a truly green solvent. The main limitation for industrial application of SCFs is related with the cost of high-pressure equipment [15].

The high catalytic efficiency of porous chemical catalysts (e.g., zeolites) in SCFs has been widely reported, especially when their active sites are placed within the internal structure of the catalyst. In that case, the high mass transfer of reactants within a catalyst’s porous structure is particularly important for increasing catalysis rates [16]. Additionally, many of the reports on SCFs catalysis mention that catalyst lifetime can be prolonged by eliminating undesired poisoning substances. As an example, the alkylation of benzene with ethene on Y-type zeolites (250–285 °C, 7–8 MPa) in scCO_2_ revealed a much lower catalyst deactivation, higher reaction rates, and higher selectivity. The reduction of catalyst deactivation was attributed to the removal of polyaromatics, considered to be precursors of coke, from the surface of the zeolites by increased solubility and diffusion rates. Extending catalyst lifetimes using SCF carbon dioxide reaction media, apart from the convenience of keeping the catalytic system running longer, also provides other potentially important economic benefits [16]. For example, due to its low viscosity and high diffusivity, scCO_2_ affords an effective and continuous cleaning within the porous catalyst structures. This good performance of heterogeneous chemical catalysts under supercritical conditions has been observed in many catalytic reactions, e.g., Fisher-Tropsh, hydroformylation, hydrogenation, Heck, Suzuki, oxidations and alkylations, even including continuous operation [17].

As regards biocatalysis, proteins are noticeably insoluble in all SCFs, allowing easy recovery and reuse, while the gas-like diffusivity and low viscosity of SCFs enhance reactant mass-transport rates to the active site of enzymes [18]. Lipases and esterases in scCO_2_ are the most widely studied systems, due to the catalytic promiscuity of these enzymes towards hydrophobic substrates and the ability of this SCF to dissolve and transport hydrophobic compounds [19]. The synthesis of aliphatic esters of different alkyl chain lengths by esterification and/or transesterification (e.g., by alcoholysis, acidolysis or inter-esterification) is a common approach to the production of terpenic flavour compounds (e.g., geranyl acetate) [20], and the valorisation of oils and fats (e.g., enrichment of acylglycerols with polyunsaturated fatty acids [21]). Currently, the use of lipases for the asymmetric synthesis of esters is one of the most important tools for organic chemists, whereby the combination of the unique properties of scCO_2_ with the catalytic excellence of lipases allows the kinetic resolution (KR) or (dynamic) kinetic resolution (DKR) of a large number of racemates (e.g., 1-phenylethanol [22], glycidol [23], *etc.*). However, it must be taken into account that CO_2_ cannot be considered completely inert with regards to its interaction with proteins. The CO_2_ forms carbamates with ε-amino groups of lysine residues placed on the enzyme surface, and lowers the pH of the aqueous layer around the enzyme, which can result in a significant decrease in the enzyme activity [24,25].

**Figure 2:**
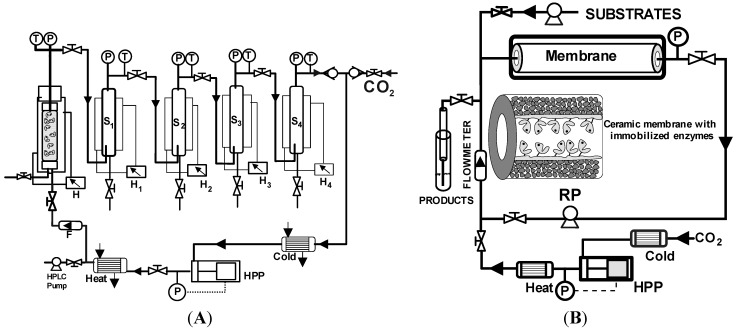
(**A**) High-pressure packed bed enzyme-reactor with recirculation, equipped with high pressure pump (HPP), separators (S), heaters (H), pressure (P) and temperature control (T), and flowmeter (F) for Lipozyme^®^-catalyzed ethyl oleate synthesis in scCO_2_ [26]; (**B**) High-pressure enzymatic membrane reactor with recirculation (B) for biotransformations in scCO_2_ [27].

The appropriate design of SCF (bio)reactors involves controlling mass-transfer limitations, environmental conditions (pressure and temperature) and product recovery. For example, Marty *et al.* [26] developed a recycling packed bed enzyme reactor at pilot scale for Lipozyme^®^-catalyzed ethyl oleate synthesis through oleic acid esterification with ethanol in scCO_2_ [see Figure 2(A)]. The proposed system assembled four high-pressure separator vessels, where a pressure cascade was produced by back-pressure valves, allowing a continuous recovery of the liquid product at the bottom of each separator, and then the recycling of the non-reacted substrates. Alternatively, membrane reactors constitute an attempt to integrate catalytic conversion, product separation and/or concentration and catalyst recovery into a single operation unit. Thus, enzymatic dynamic membranes, formed by depositing water-soluble polymers (e.g., gelatine, polyethyleneimine, *etc.*) on a ceramic porous support, exhibited appropriate properties for continuous butyl butyrate synthesis in scCO_2_, along with a high operational stability [27,28].

The most important environmental factors affecting (bio)catalyst performance in SCFs are pressure, temperature and water content. Indeed, these parameters influence all mass-transfer phenomena and (bio)catalyst activity by changing the rate-limiting steps or modulating their selectivity [29,30]. As an example, the catalytic activity of immobilized lipase for butyl butyrate synthesis was exponentially increased by the drop in scCO_2_ density accompanying different combinations of pressure and temperature [27]. In the same context, for the pressure range of 7.7 to 8.5 MPa, the changes in the conformation of lipase resulted in an increased rate of esterification reactions. Thus, Dhake *et al*. obtained good yields (up to 99%) for the enzymatic synthesis of several citronellyl esters at 45 °C and 8.0 MPa [31]. Temperature influences enzyme activity much more than pressure not only due to the usual increment in reaction rates at higher temperatures, but also because of enzyme thermodeactivation processes. The optimal temperature of enzymatic processes in SCFs is related with pressure as both control solvent properties [29]. Water concentration also greatly influences enzyme activity because proteins require a specific amount of bound water molecules to be active. When the water content is too high or if the water is a product of the reaction in the supercritical medium, the resulting increased humidity may lead to enzyme deactivation. Thus, the actual amount of water needed is specific to each SCF-substrate-enzyme system, and must be maintained constant throughout the process [32]. 

However, all the unique properties of scCO_2_ in its role as green solvent to extract, dissolve and transport chemicals are tarnished by its denaturative effect on enzymes. Several approaches have been developed to protect enzymes against these adverse effects of scCO_2_, including covalent attachment on supports coated with hydrophilic polymers, the entrapment of enzymes in silica-aerogels, and the use of cross-linking enzyme aggregates. In spite of the advantages obtained with all these stabilization approaches, the best results for enzyme-catalyzed reactions in scCO_2_ were observed when the biocatalyst was applied in suspension or coated with other green solvents, such as ionic liquids, as described below [19].

There are additional challenges and/or weakness that need to be addressed before SCFs technology can play a major role in industrial applications. Those include its cost, energy consumption, and safety issues in the operation process. The SCFs technology requires expensive equipments, such as strong durable reactors, high pressure pumps, efficient control devices, *etc.*, and the costs involved in operation and maintenance are also higher than those of conventional processes. Furthermore, the energy consumption to reach supercritical conditions is dependent of the nature of the solvent in question (see Table 1 for critical parameters), and this may be unsustainable for long term industrial application. An example is the biodiesel synthesis by non-catalytic supercritical alcohol technology requiring temperatures up to 200 °C (the critical temperature of methanol is 239 °C). Thus, we must bear in mind that the energy utilized in the manufacturing process should be not exceed the energy provided by this biofuel [33]. Regarding the safety of SCFs processes, they are usually labelled as high risk processes because of the combination of high pressures and temperatures. The SCFs technology for the industrial production of biodiesel could be improved by the design of small reactors with enhanced catalytic efficiency, which, in combination with continuous operation approaches, may help offset the described weaknesses.

## 3. Non-catalytic Biodiesel Synthesis by Supercritical Fluid Technology

### 3.1. General Considerations

Product separation and isolation along with catalyst recovery are by far the most energy intensive steps in biodiesel production and, therefore, the most economically unfavorable. Supercritical fluid technology may provide some advantages, facilitating these separation and isolation processes. The use of a supercritical fluid phase as solvent/reagent for biodiesel synthesis seems, accordingly, a straightforward approach. Taking into account that the nature of the solvent is controlled by the pressure and temperature, the formation of a single phase between the reagents and the triglycerides can be induced. Although high pressures (>5 MPa) and temperatures (>200 °C) are needed to reach a homogeneous phase, the transesterification reaction kinetics is favoured under these harsh conditions. Indeed, the reaction can proceed in the absence of any catalyst, leading to non-catalytic biodiesel synthesis. Furthermore, the downstream isolation of the biodiesel from glycerol is simpler since many discrete operations such as catalyst neutralization and separation are not required, leading to products of higher purity, which is also important from a economic point of view. Thus, SCFs technology, even though it requires the use of high temperatures and pressures, may provide distinct advantages in comparison over conventional technologies. For instance, in the conventional alkali-catalyzed method, the final glycerol phase contains methanol, water, alkaline catalyst and soap. Thus, the selling price value of this crude glycerol is extremely low compared with the purified glycerol commonly used in food and pharmaceutical products [34]. 

**Figure 3:**
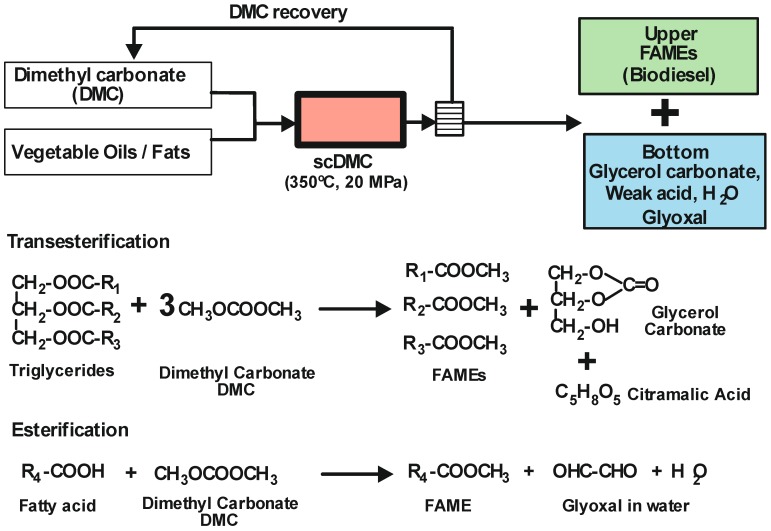
Non-catalytic one-step process for biodiesel synthesis using supercritical dimethyl carbonate. Reaction equations.

The thermal stability of fatty acid methyl esters and biodiesel prepared by non-catalytic SCFs methods and obtained from various plant oils has been studied over a wide range of high temperatures (270–430 °C) and pressures (17–56 MPa) in order to evaluate the effect of the harsh experimental conditions on the biodiesel fuel quality [35,36]. It was found that although all fatty acid methyl esters, including the poly-unsaturated ones, are stable at low temperatures and pressures, but they partially decomposed with isomerization from *cis*-olefin to *trans*-olefin at high pressure and temperature. From these results, it was suggested that, for high-quality biodiesel production, the reaction temperatures in SCFs processes should be maintained below 300 °C, preferably at 270 °C for supercritical methanol (scMeOH), and lower than 360 °C for supercritical methyl acetate (scMeOAc) with a supercritical pressure higher than 8.09 MPa (see Figure 3). Besides, higher temperatures increase energy consumption and thus operating costs. However, it has been suggested that by selecting the adequate experimental conditions, processing could use less energy than the conventional method. Justification for this claim was based on the absence of the need for catalyst addition and energy intensive purification operations (distillation), making this technology economically competitive with the conventional ones [37]. As regards reactor design, although batch biodiesel production is favoured over continuous processes in many research laboratories and in large scale operations, continuous operation is expected to gain wider acceptance, considering their added advantages that include a higher production capacity and lower operating costs to ensure the long term supply of biodiesel. Additionally, key parameters such as oil concentration in the supercritical phase, and the density of the mixture (depending on temperature, pressure and composition) can be easily controlled and optimized under flow conditions [38,39,40,41].

Alcohols are the most obvious choice for the development of non-catalytic biodiesel synthesis by transesterification reactions in SCFs [42]. Both ethanol and, especially, methanol, have been reported as supercritical fluids for biodiesel synthesis [43,44], although other fluids in supercritical conditions (e.g., dimethyl carbonate, methyl acetate) have also been evaluated [45]. 

### 3.2. Single Step SCFs Processes for Non-catalytic Biodiesel Synthesis

#### 3.2.1. Supercritical Alcohol

Supercritical methanol (scMeOH), whose critical parameters are T_c_ = 239.4 °C, P_c_ = 8.1 MPa, and ρ_c_ = 0.276 g·cm^−3^, was one of the first SCFs evaluated for the production of biodiesel [46]. In light of the promising results obtained with supercritical alcohols, many researchers have studied the effects of various reactions parameters on FAMEs yield. As mentioned, using SCFs it is possible the tuning and control of the solvent polarity through the adjustment of temperature (T) and pressure (P). Indeed, the dielectric constant (ε) of methanol decreases from 34 to 5 upon isobaric heating from 25 to 260 °C at 20 MPa [47]. The hydrogen bond network existing in liquid methanol is broken under SC conditions, leading to a fluid in which only small oligomers exist [48,49]. Reduction of the polarity and the hydrogen bonding can facilitate a much stronger direct nucleophilic attack by the methanol on the carbonyl carbon rendering a catalyst unnecessary. Furthermore, reduced dielectric constant may even lead to a single oil/alcohol phase, increasing the reaction rate [50]. These facts explain the differences observed when the reaction is performed either under sub-critical or supercritical conditions since the reaction kinetics is highly dependent on the homogeneity of the reaction mixture. It seems that operating under supercritical conditions enhances the rate of reaction [51]. The solubility of reactants and transesterification products (homogeneous or heterogeneous) in the alcohol under various pressure-temperature-molar fraction (P-T-x) conditions is a keystone for rapid and complete tranesterification reactions [42]. Liquid-vapor-SC phase transitions for the binary/ternary systems (soybean oil and alcohol at different oil:ethanol molar ratio) were studied by Anitescu *et al* using a view cell attached to the outlet of the reactor, which was heated from 26 °C to 400 °C at constant volume/density [52]. With two liquid phases in ambient conditions, the system showed increasing homogeneity with increasing temperature and pressure until a single SC phase was reached. Under these conditions, the conversion of triglyceride to FAMEs was very rapid compared to that observed under subcritical conditions [53,54]. The reaction in continuous flow conditions yields above 98% conversion, with a single clear phase at moderate pressures (10–20 MPa), high temperatures (375–400 °C), low methanol ratios (3:1–6:1) and short reaction times (<3 min). These results are in contrast with those reported by Brignole and co-workers, who observed that high conversion can be obtained, in most cases, even when operating in the two-phase region [55]. In this case, the oil transesterification could mainly occur in the light supercritical phase, where the oil and the monoglycerides and diglycerides are partially soluble and the concentration of methanol is high. Moreover, the light-phase transport properties favour a higher reaction rate. The increased methanol/oil ratio leads to a homogeneous supercritical phase by increasing the system temperature while working at moderate pressures of 10–15 MPa, since an increase in the methanol/oil ratio decreases the critical temperature of the system [56,57]. Although a high methanol ratio favours the formation of a homogeneous supercritical phase while also driving the reaction to completion, it also increases costs and energy consumption. The addition of a co-solvent such as propane or CO_2_ decreases the critical point of the mixture, allowing the reduction of both temperature and the oil/alcohol ratio [58,59,60,61].

An additional advantage of the use of supercritical alcohols in biodiesel synthesis is the high tolerance towards possible feedstock contaminants, mainly FFAs and water. In supercritical alcohols, esterification of the FFAs to the corresponding fatty acid alkyl esters takes place simultaneously to oil transesterification, yielding higher amounts of biodiesel. Thus, low cost feedstock, usually containing high portions of FFA/oil, can be used in the process without any additional pretreatment. As regards the water content, transesterification reactions catalyzed under acid or alkaline conditions are highly sensitive to the presence of water as hydrolysis of the corresponding esters are favoured in the presence of water. In contrast, the non-catalytic supercritical process can be performed even with a high water contents [62].

#### 3.2.2. Supercritical Dimethyl Carbonate (scDMC)

It has recently been demonstrated that biodiesel can be produced from triglycerides and dimethyl carbonate instead of methanol, in a non-catalytic process by using supercritical dimethyl carbonate (T_c_ = 274.9 °C, p_c_ = 4.6 MPa) [63,64,65]. In this way, triglycerides as well as fatty acids are successfully converted to fatty acid methyl esters (FAMEs). Besides, with this methodology another valuable compound, glycerol carbonate, is obtained as a secondary product, instead of the undesirable glycerol, and a weak acid, such as citramalic acid, as the main by-product (see Figure 3). Glycerol carbonate has a higher commercial value compared than glycerol and has a greater potential for industrial application [66]. Hence, this methodology could have a large impact on biodiesel economics as two high added value products are produced in a single-step reaction, leading to increases cost effectiveness, even if high temperatures and pressures are required. The FAMEs produced by this method are obtained at higher yields than with supercritical methanol, and satisfy the international standards for use as biodiesel fuel. For instance, up to a 97.4 wt% yield can be obtained for the scDMC method at 300 °C, 20 MPa, 20 min and a 42:1 molar ratio of dimethyl carbonate to oil [67]. The properties of the biodiesel produced by this method have been evaluated according to different international standards. Overall, the FAMEs from the scDMC method satisfied all the requirements for international biodiesel standards except the specification regarding oxidation stability, which is an important parameter for preventing deterioration. However, this problem could be diminished by the addition of an antioxidant or by using oils containing lower levels of unsaturated fatty acids [68].

#### 3.2.3. Supercritical Carboxylate Esters

**Figure 4:**
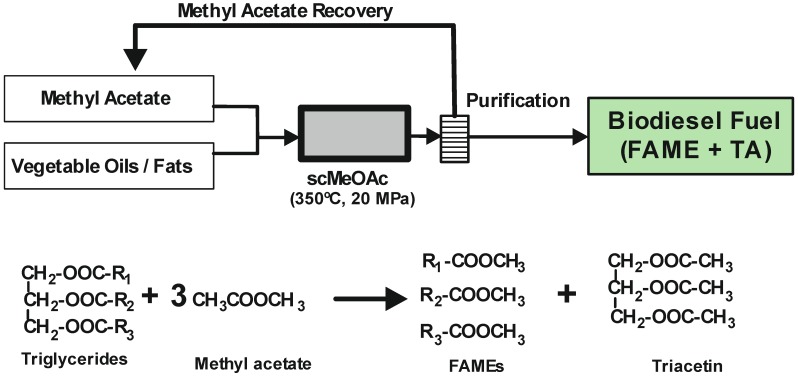
Biodiesel fuel synthesis using supercritical methyl acetate (scMeOAc). Reaction equations.

In order to prevent the production of glycerol as a by-product, a process was designed using methyl acetate instead of methanol under supercritical conditions [69]. The supercritical methyl acetate (scMeOAc) non-catalytic method converts triglycerides into fatty acid methyl esters (FAMEs) and triacetin, instead of glycerol. Furthermore, it has been discovered that there were no adverse effects on the main fuel characteristics when the molar ratio of methyl oleate to triacetin was 3:1, which corresponds to the theoretically derived mole ratio from the trans-esterification reaction of rapeseed oil with methyl acetate (see Figure 4). Moreover, the addition of triacetin to methyl oleate improved the *pour point* (defined as the lowest temperature at which a fuel product will begin to flow), and triacetin has a high oxidation stability. Therefore, by defining biodiesel fuel as a mixture of methyl oleate and triacetin, the supercritical methyl acetate can lead to an improved yield (105%) of biodiesel fuel compared with the conventional process. However, this value is lower than the maximum recovery expected (125% yield) because of the breakdown at the required high temperatures of some unsaturated fatty acid methyl esters. Despite this, the scMeOAc method not only improves the quality of the biodiesel fuel but also minimizes the separation and purification steps, and therefore the energy required. Saka *et al.* [70] have used various supercritical carboxylate esters to convert triglycerides into FAMEs and triacetin in the absence of any catalyst. The highest product yield was obtained with supercritical methyl acetate, 97.7 wt% considering a mixture of both FAMEs and triacetin as biodiesel fuel. The lower reactivity shown by other carboxylate esters seems to be related to the length of alkyl chains in the esters, e.g., the longer alkyl chains in both alcohol and acyl moieties of carboxylate esters provides the lowest product yields [71]. Banchero and co-workers [72] demonstrated that supercritical methyl acetate can be successfully used in the transesterification process of different vegetable oils to obtain biodiesel fuel. The process was suitable for edible, non-edible or waste oils, regardless of the FFA content which usually affects the conventional transesterification process at atmospheric pressure. All the examined oils reached complete conversion after 50 min at 345 °C, 20 MPa and with a methyl acetate:oil molar ratio of 42:1. 

### 3.3. Two-step SCFs Process for Non-Catalytic Biodiesel Synthesis

An alternative non-catalytic process in supercritical fluids for the synthesis of biodiesel fuel consists in a two steps process. In this method, triglycerides are firstly hydrolyzed to FFAs, which, in a second consecutive step, are esterified to the corresponding esters.

**Figure 5:**
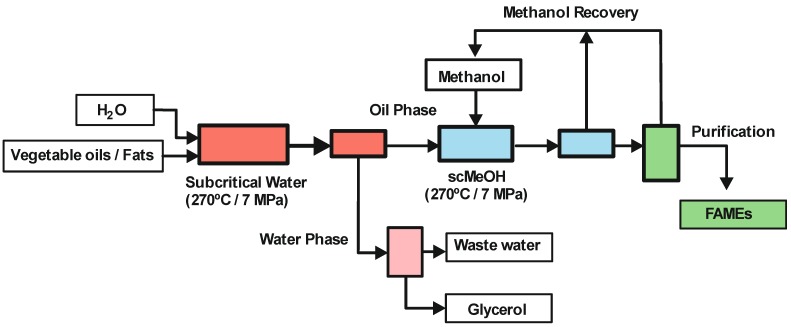
Biodiesel synthesis by the Saka-Dadan two-step supercritical method [74].

The first example developed according to this concept is known as the “Saka-Dadan” SCF method. In the first step the unique properties of scH_2_O are exploited. It is well known that the dielectric constant of water decreases from ε = 80 at standard T and P to ε = 31 at 225 °C, P = 10.0 MPa, and finally to ε = 6 at the critical point, *Tc* = 374.15 °C, *Pc* = 22.1 MPa, due to the steady decrease in the effectiveness of the hydrogen bonds with increasing temperature [73]. Thus, the reduction of the dielectric constant with increasing temperature promotes the miscibility between water and the oil, favouring a hydrolysis process. Furthermore, at a constant pressure of 25 MPa, the ionic product of water increases with temperature until it reaches a maximum (10^−11^) at 250 °C. This allows acid- and base-catalysed reactions to be performed in high temperature pressurized water with no catalyst. Hence, scH_2_O is used as both the solvent and the catalyst for the hydrolysis step at 270 °C, 7 MPa and a volumetric water/triglyceride ratio of 1:1 [74]. These conditions are milder than those reported for the original one step in scMeOH (350 °C and 20–50 MPa), thus reducing the energy consumption. After hydrolysis, two layers are formed, the upper portion containing fatty acids and the lower portion water with glycerol. In a second step, FAMEs are produced in supercritical methanol after 20 min at 270 °C and 7 MPa (see Figure 5).

Alternatively, a two-step supercritical dimethyl carbonate method, named the “*Saka and Ilham Process*” has been proposed. This consists of the hydrolysis of triglycerides to fatty acids in subcritical water (270 °C and 27 MPa) followed in a second step by the esterification of fatty acids to FAME in supercritical dimethyl carbonate (300 °C and 9 MPa) [65]. Various parameters affecting the yield of fatty acids and FAMEs have been studied and a production scheme has been proposed. The supercritical dimethyl carbonate method is applicable to oils with a high free fatty acid contents such as *Jatropha curcas* oil, with 13.6 wt% free fatty acids. The glycerol produced in the first step was valorized in the second step, obtaining glycerol carbonate in supercritical dimethyl carbonate (280–300 °C and 9–12 MPa) without any catalyst [75]. The glycerol showed higher rate of conversion to glycerol carbonate due to its high purity. This glycerol conversion process was coupled with hydrolyzed fatty acids conversion into FAMEs to establish the two-step supercritical dimethyl carbonate method (Saka and Ilham Process). The non-catalytic two-step supercritical dimethyl carbonate method is a good process to produce high yields of FAMEs, even from oils with high levels of unsaturated fatty acids, together with a value added by-product, glycerol carbonate, under non-acidic and mild reaction conditions [68].

**Figure 6:**
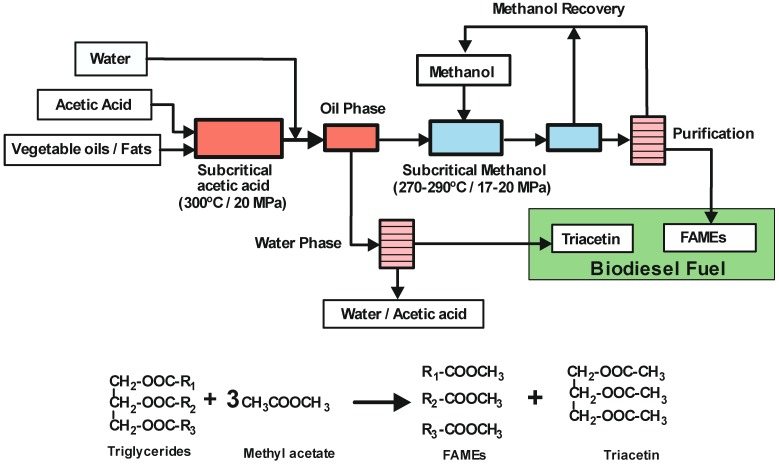
Biodiesel synthesis by the two-step supercritical approach. Reaction equations [76].

The same group has also reported a “two-step” process, in which first step is a transesterification of the oil to triacetin carried out in either subcritical or supercritical acetic acid, followed by supercritical methanol treatment. A transesterification reaction occurs between acetic acid and the triglycerides under subcritical conditions without the addition of catalysts. It was clear that fatty acids and triacetin could be obtained at high yields (see Figure 6). The reaction rate in acetic acid was much higher than when either methyl acetate or methanol was used in the transesterification step. The yield of triacetin recovery from subcritical acetic acid treatment by aqueous washing was 64%. On the other hand, when the oil phase from the previous treatment containing fatty acids was supplied to the supercritical methanol process at 270 °C and 17 MPa for 15 min, a 97 wt% yield of FAMEs was obtained. The recovery rate of biodiesel fuel from this process was 117wt%, a higher value than that obtained by conventional biodiesel fuel manufacturing processes, which produce glycerol as a by-product. This recovery rate is also higher than for the recently reported supercritical methyl acetate method (*Saka and Isayama method*) [76]. 

## 4. Biocatalytic Synthesis of Biodiesel in Supercritical Fluids

The use of dense gases, mainly scCO_2_ or propane, as alternatives to organic solvents, to provide efficient reaction media for the enzymatic synthesis of biodiesel might be considered as the cleanest approach. As indicated before, the non-miscibility between triacylglycerides and methanol starting substrates, and glycerol and biodiesel final products, is rate-limiting because of the low efficiency in mass-transfer phenomena during the process, as well as the loss of activity of the biocatalyst through direct contact with methanol and glycerol. SFCs have shown to be suitable systems to overcome these drawbacks following the appropriate design of both reactor and reaction conditions.

Investigation of the phase behaviour of the reaction system is important for better understanding the enzymatic synthesis of biodiesel in supercritical fluids. Pressure and temperature can affect the reaction rate by affecting the partitioning of the reaction components between the two phases, leading to poor conversions at the enzyme surface. Thus, Ciftci *et al.* studied the phase behaviour of corn oil-methanol-CO_2_ mixtures as a function of pressure by using a phase equilibrium cell equipped with a sapphire window [77]. Visual observation of the phase behaviour of the reaction mixture upon pressurization at 55 °C showed that the immiscible biphasic liquid phase, consisting of corn oil and methanol, approached a single liquid phase at pressures above the critical pressure of CO_2_ and also expanded through the dissolution of CO_2_. However, as a single homogeneous phase was not fully formed at the pressure levels investigated in this study (11.0, 20.0 and 35.0 MPa), authors consider reaction mixture as a two phase (lower single liquid phase and upper CO_2_ phase) system. Other authors studied the solubility of olive husk oil in scCO_2_ (40–80 °C, 15.0–35.0 MPa), and the influence of methanol and ethanol (1–5% v/v) as cosolvents, by using a dynamic flow method. It was observed how the solubility of the triglyceride increased moderately when increasing the CO_2_ pressure at constant temperature, but doubled when 5% (v/v) of cosolvents was added [78]. The phase behaviour of the CO_2_-biodiesel system in the presence of cosolvents (e.g., methanol [79], ethanol [80], *etc.*) has also been studied, leading to the observation that the addition of alcohol improves their miscibility.

The design of the supercritical reactor used has been seen to be key element for the efficiency of the (bio)catalytic synthesis of biodiesel. Both continuous and discontinuous reactors have been assayed using several types of immobilized lipases as biocatalysts, and optimized reaction conditions (pressure, temperature, cosolvents, stirring, *etc.*) and yields near to 100% biodiesel have been achieved. As an example of the use of discontinuous reactors, Lee *et al.* [81,82] carried out biodiesel synthesis in scCO_2_ using a mixture of vegetable oil and methanol (1:4 molar ratio) and a mixture of immobilized of *Candida rugosa* and *Rhizopus oryzae* lipases, as biocatalysts. In these studies, various factors, such as temperature, pressure, stirring speed, and the concentration of immobilized enzymes were investigated, resulting in optimal conditions for biodiesel production (100% yield at 2 h) under the following conditions: 13.0 MPa pressure, 45 °C temperature, 250 rpm stirring speed, and 20% (w/w) immobilized enzyme. Furthermore, an optimized stepwise addition of methanol allowed for the maintenance of immobilized lipase activity and the recycling of the immobilized biocatalysts. The yield of these stepwise reactions was still 85% after 20 reuses.

The usual advantages of continuous processes are even more evident in the case of the enzymatic synthesis of biodiesel because products are directly separated, and the methanol concentration has a low impact on the enzyme deactivation [13,17]. Jackson *et al.* [83] were pioneers in the applicability of scCO_2_ in the continuous enzymatic synthesis of biodiesel from soybean and corn oils with reaction conversions above 98% at 1 mL/min CO_2_ flow rate, followed by complete fractionation of the reaction mixture. In order to improve the efficiency of the continuous process for biodiesel synthesis in scCO_2_ using immobilized enzymes as catalysts, several authors described how the appropriate design of the continuous reactor may provide operational advantages (e.g., efficient mixture of substrates, continuous separation of products, enhanced operational stability of enzymes, *etc.*) [77].

**Figure 7:**
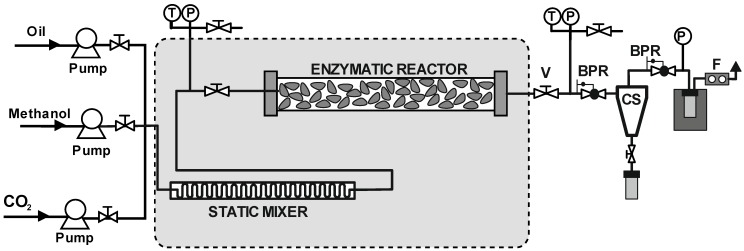
Set-up of the continuous enzymatic reactor for biodiesel synthesis [84]. T: temperature indicator; P: pressure indicator; V: valve; BPR: Back-pressure regulator; CS: cyclone separator; F: Flow.

Barreiros and co-workers [84] implemented a continuous process for biodiesel production in supercritical carbon dioxide by using virgin sunflower oil and methanol as substrates, which were incorporated in the reactor using a static mixer with 27 elements (See Figure 7). The application of Lipozyme TL IM as biocatalyst led to FAMEs yields that exceeded 98% at 20.0 MPa and 40 °C, for a residence time of 20 s and an oil to methanol molar ratio of 1:24. Even for moderate reaction conversions, a fractionation stage based on two high pressure cyclone separators provided FAMEs of up to 96% purity. In the same context, Dalla Rosa *et al* [85] reported the continuous production of fatty acid ethyl esters from soybean oil in compressed carbon dioxide, propane and *n*-butane, using immobilized Novozym 435 as the catalyst. The experiments were performed in a packed-bed bioreactor. An evaluation of the effects of pressure and temperature in the range of 5.0 to 15.0 MPa and 30–70 °C, respectively, as well as the oil to ethanol molar ratio (from 1:6 to 1:18) and solvent to substrates mass ratio (from 4:1 to 10:1) was carried out. The best results (up to 98% yield) were obtained for lipase-catalyzed alcoholysis in a continuous tubular reactor using compressed propane (70 °C, 6.0 MPa), which it is suggested as another potential route to biodiesel production. The similar dielectric constants of compressed propane and carbon dioxide, together with the higher pressure phase transition values generally found in systems formed by carbon dioxide and high molecular weight compounds (e.g., triglycerides)lead weight to the belief that propane may also be suitable as reaction medium for enzyme-catalyzed bioconversions [29,86].

Biphasic systems based on ILs and scCO_2_ have recently been used for the enzymatic synthesis of biodiesel [87]. For example, a continuous enzymatic reactor, based on supported biocatalyst particles coated with hydrophobic ILs having imidazolium cations with C_12_-C_18_ alkyl side chains was studied for the methanolysis of triolein in scCO_2_ at 60 °C and 18.0 MPa. The operational stability of the immobilized lipase was improved by its coating with the ILs, *i.e.*, 1-methyl-3-octadecylimidazolium hexafluorophosphate. A final two-phase system was obtained, revealing a good catalytic behaviour in continuous operation under supercritical conditions, with up to 82% biodiesel yield after 12 cycles of 4 h. For these IL/scCO_2_ systems, the unique properties of long chain ILs, providing a very appropriate microenvironment for enzyme-catalyzed reactions, led to a clear improvement in the efficiency for the biotransformation of vegetable oils into biodiesel. 

However, the full miscibility of these hydrophobic ILs with both the triolein substrate and the methyl oleate product was related with the continuous activity decay observed for long term operation cycles. Thus, an excess in the triolein inlet flow or the continuous release of biodiesel product from the enzyme particle to the scCO_2_ flow may dissolve the protective IL shell, enhancing continuous enzyme deactivation. Similarly, the low efficiency of the hydrophobic scCO_2_ phase in transporting the hydrophilic by-product glycerol could led to its retention in the closest enzyme microenvironment and to the continuous biocatalyst poisoning, preventing the entry of new triolein substrate molecules. Different experimental approaches to efficiently desorb glycerol from the catalytic matrix in biodiesel synthetic processes under supercritical conditions have been proposed. Desorption was carried out using absolute ethanol under atmospheric conditions at different mass flows (10–30 g/min) or using ethanol-modified supercritical CO_2_ (1:3 molar ratio of ethanol:CO_2_), under a pressure of 14.0 MPa, within a temperature range of 106–134 °C and with mass flow rates of 6–34 g/min. The results showed that ethanol is an efficient solvent for this process and that the supercritical desorption is much faster than conventional desorption processes [88].

The development of solid supports with a covalently attached IL phase is a further step towards reducing the amount of ILs used in catalytic processes in scCO_2_, providing permanent protection of the enzymes against the adverse effect of this SCF. Through this approach, the properties of ILs are transferred to the solid phase, leading to either particle- or monolithic-Supported Ionic Liquid-Like Phases (SILLPs) [89]. This kind of material with tunable properties can be prepared on demand and they have been shown to efficiently immobilize a variety of catalysts. Indeed, the microenvironment provided by these “solid ionic liquid phases” can be modified by the modulation of the different design vectors of SILLPs [90]. Bioreactors based on *Candida antarctica *lipase B (CALB) adsorbed onto SILLPs have been successfully applied as macroporous monolithic mini-flow systems for the continuous synthesis of citronellyl propionate in scCO_2_ [91], as well as the KR and DKR of *rac*-1-phenyhlethanol [92], showing in both cases good enzymatic activity and operational stability.

**Figure 8:**
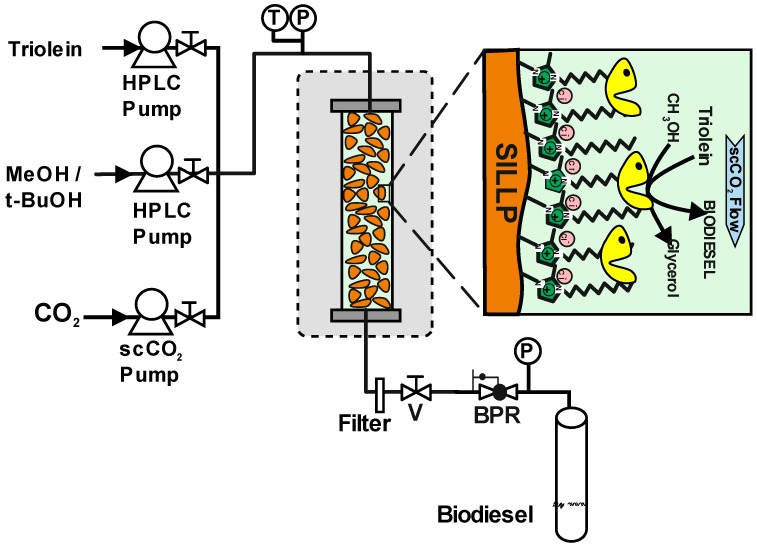
Set-up of a continuous reactor containing immobilized lipase onto Supported Ionic Liquid-Like Phases (SILLPs) for biodiesel synthesis by methanolysis of triolein [93]. T, Temperature indicator; P, pressure indicator; V, valve; BPR, Back-pressure regulator.

In this context, the use of different nano-structured supports, based on different 1-decyl-2-methylimidazolium cations covalently attached onto a polystyrene-divinylbenzene (PS-DVB) porous matrix as carriers to immobilize *Candida antarctica* lipase B (CALB), was recently reported (see Figure 8) [93]. The suitability of these immobilized lipase derivatives to carry out the synthesis of biodiesel (methyl oleate) through methanolysis of triolein has been tested in both *tert*-butanol and supercritical carbon dioxide (18.0 MPa, 45 °C) as reaction media. The use of modified supports with low ionic liquid loads covalently attached to the main polymeric backbone chains provided structured materials that led to high biodiesel yields (up to 95%) and operational stability (85% biodiesel yield after 45 cycles of 8–4 h) in scCO_2_ (45 °C, 18.0 MPa). The presence of *tert*-butanol, as an inert co-solvent, in the scCO_2_ phase at the same concentration as triolein was the key to avoid the continuous poisoning of the biocatalyst through the blocking of its active sites by the polar by-product (glycerol) produced in the biodiesel synthesis. These results clearly illustrate the potential of SILLP-supported biocatalysts for the production of biodiesel, which can be obtained by means of a fully green technology under continuous operation.

## 5. Future Trends

In this review, we have highlighted the enormous potential of applying supercritical fluid technologies to design processes of biodiesel fuel production. However, as for any emerging technology, it also presents some weaknesses. These are challenging and should be solved before the industrial implementation of such methodologies. Our future efforts should move forward to simpler, cost-effective and “greener” SCFs processes, leading to high yields of a quality biodiesel fuel. To achieve this goal, new non-edible potential sources of vegetable oil such as *microalgae* and *jatropha* oil, which ensure that biodiesel production does not compete with resources for food industry, should be taken into consideration, as they will help to reduce costs and increase social acceptance. One of the most important approaches for the design of more intensive and cost-effective process configurations is process integration. In this respect, SCFs technology can help to process integration by combining routes for the simultaneous extraction, transesterification and valorisation of glycerol in a single step. Such technology should combine chemical reactions and extraction in the same step, achieving synergistic effects, and leading to an increase in selectivity, conversion, productivity and purity of the final product(s). Simpler reactive extraction processes without or with the (bio)catalysts can be a potential route for biodiesel production, greatly reducing the processing steps and costs at the same time. 

The extreme reaction conditions necessary when working with SCFs make any such process energy intensive. This can be counterbalanced by the improvement in mass and heat transfer intensified by the fast fluid flow conditions, hence decreasing mass-transfer limitations, and avoiding phase separation, as a consequence of the large surface area-to-volume ratio available. The use of continuous-flow/micro(mini)reactor technology improves heat and mass transfer. This makes possible the potential for higher conversion yields under milder conditions and involving reduced molar ratios of alcohol to oil, as well as lower reaction temperatures and (bio)catalysts concentrations than for conventional stirred reactors. The development of new, simple, cheap and environmentally friendly catalytic systems for biodiesel production is required. Additional studies into the economic viability of any of the approaches mentioned should be made before we can safety talk of a green chemical industry for biodiesel production.
